# Aortic Bioprosthetic Stenosis Leading to Heyde Syndrome

**DOI:** 10.1016/j.jaccas.2025.105828

**Published:** 2025-10-23

**Authors:** Ahad Firoz, Justin Devera, Matthew Lam, Yves-Paul Nakache

**Affiliations:** a Department of Internal Medicine, University of California–Davis Medical Center, Sacramento, California, USA; b Division of Cardiovascular Medicine, Section of Internal Medicine, University of California–Davis Medical Center, Sacramento, California, USA; c Division of Cardiovascular Medicine, Sacramento Veterans Affairs Medical Center, Mather, California, USA; d Department of Hospital Medicine, Sacramento Veterans Affairs Medical Center, Mather, California, USA

**Keywords:** aortic valve, cardiovascular disease, echocardiography, hemorrhage, murmur, physical examination, stenosis, valve repair, valve replacement

## Abstract

**Background:**

Heyde syndrome (HS) is the constellation of aortic stenosis and gastrointestinal bleeding due to arteriovenous malformation (AVM).

**Case Summary:**

A 74-year old man presented with angina, dyspnea, and fatigue. His work-up was pertinent for significant anemia, severe bioprosthetic aortic stenosis, and a duodenal AVM. The patient required blood transfusions and AVM clipping. He was diagnosed with bioprosthetic HS and referred to the cardiology team for transcatheter aortic valve replacement consideration.

**Discussion:**

Reports of bioprosthetic HS are scarce in the literature. This case report describes the investigation and management of HS amid recurrent anemic episodes and diagnostic uncertainty.

**Take-Home Messages:**

Recurrent episodes of idiopathic anemia in patients with a prosthetic valve should raise concern for HS. Clinicians with a high suspicion of HS and negative esophagogastroduodenoscopy/colonoscopy should pursue capsule endoscopy or push enteroscopy to fully assess the small bowel. A normal von Willebrand factor in isolation should not rule out HS.

## Visual Summary

**Figure:**
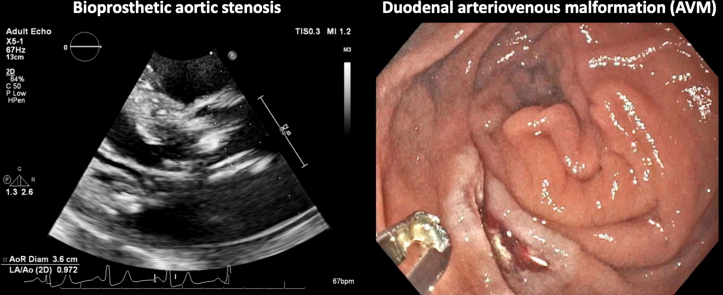

**Visual Summary:**
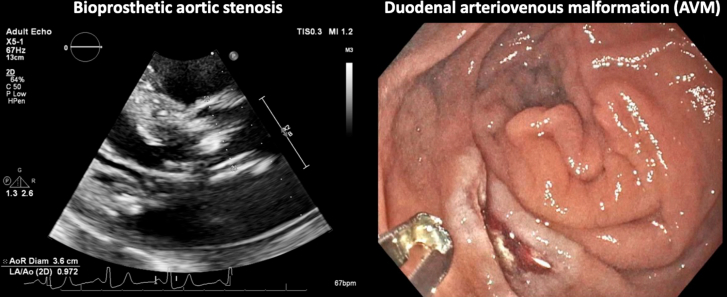
An Atypical Case of Bioprosthetic Heyde Syndrome (Left) Severe stenosis of the bioprosthetic aortic valve. (Right) An arteriovenous malformation in the duodenal bulb.

## Case Presentation

We present the case of a 74-year old man with a pertinent history of severe aortic stenosis (AS) after having undergone bioprosthetic aortic valve replacement (AVR) who was brought to the emergency department after 1 week of angina and dyspnea. Other relevant symptoms included fatigue and dark, sticky stool for the past month. Otherwise, he denied frank melena, hematochezia, hematemesis, hemoptysis, hematuria, or other sources of bleeding. He was recently started on aspirin but denied anticoagulation use.

He was hemodynamically stable on presentation and had normal oxygen saturation on room air. Physical examination was pertinent for dry mucous membranes, conjunctival and skin pallor, and a prominent systolic murmur.

**Take-Home Messages**

• Recurrent episodes of idiopathic anemia in patients with a prosthetic valve should raise concern for Heyde syndrome.
• Clinicians with a high suspicion of Heyde syndrome and negative EGD/colonoscopy should pursue capsule endoscopy or push enteroscopy to fully assess the small bowel.
• A normal von Willebrand factor in isolation should not rule out Heyde syndrome.

## Past Medical History

The patient's medical history included iron deficiency anemia, severe AS after 23-mm pericardial bioprosthetic AVR (Edwards Lifesciences model 3300TFX) in 2015, chronic obstructive pulmonary disease, seizure disorder, heart failure with preserved ejection fraction, and hypertension.

Of note, the patient had 2 previous pertinent hospitalizations. The first one occurred in 2015, during which he was noted to have a new normocytic anemia. Given his known severe, symptomatic AS, Heyde syndrome (HS) had already been suspected. However, an esophagogastroduodenoscopy (EGD) and colonoscopy were not performed. A few months later, he underwent the surgical AVR with bioprosthetic graft. Echocardiography later that year showed a well-seated prosthetic valve with normal gradients and no significant stenosis.

After his AVR, he remained in stable health until his second hospitalization in 2024. At that time, he presented to the emergency department with fatigue and weakness and was found to have a sudden drop in his hemoglobin to 3.7 g/dL. He denied any frank melena or hematochezia during this encounter. Iron studies were consistent with an iron deficiency anemia; his clinical status improved after transfusing 3 U of packed red blood cells (pRBC). HS was again the leading diagnosis on the differential. However, the patient declined EGD and colonoscopy studies, preventing a definitive diagnosis.

## Differential Diagnoses

Considerations included heart failure exacerbation, HS, and anemia secondary to upper gastrointestinal bleed (GIB).

## Investigation

**Figure 1:**
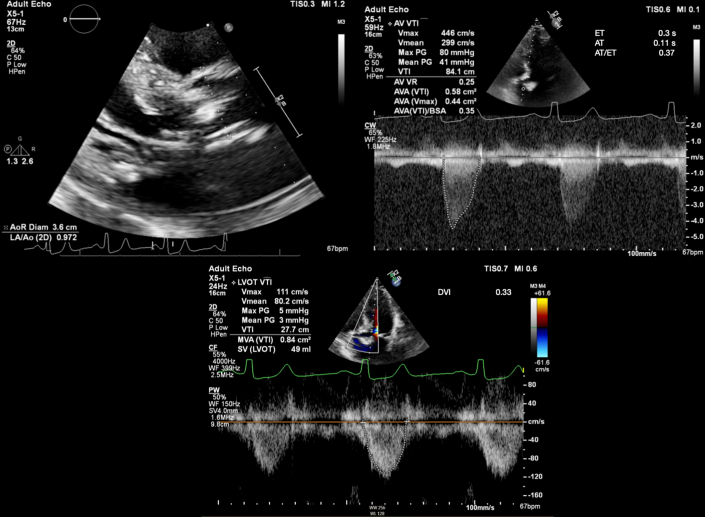
Initial Transthoracic Echocardiography Images Transthoracic echocardiography images displaying severe bioprosthetic aortic stenosis.

**Figure 2:**
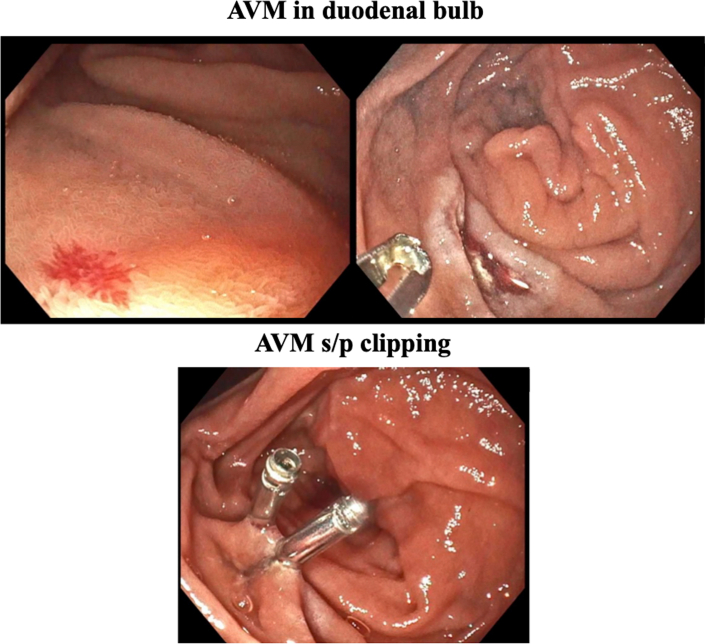
Images During EGD EGD images displaying (Top) an AVM in the duodenal bulb and (Bottom) subsequent AVM clipping. AVM = arteriovenous malformation; EGD = esophagogastroduodenoscopy.

Pertinent laboratory work-ups included a severe macrocytic anemia, with a hemoglobin of 5.5 g/dL on admission. He was also noted to have a mild, prerenal, acute kidney injury. Ischemic cardiac work-up, including electrocardiogram and high-sensitivity troponins, were negative. Coagulation, iron, and hemolysis laboratory tests were not performed before blood transfusion in the emergency department. The patient was admitted to the internal medicine service with plans to obtain an inpatient EGD and colonoscopy. Additionally, transthoracic echocardiography (TTE) was ordered to reassess his bioprosthetic valve given his prominent systolic murmur; pertinent TTE images are displayed in Figure 1 and a clip in the parasternal long-axis view is also included (Video 1). His TTE showed a well-seated bioprosthetic aortic valve with no paravalvular leak; however, there were new thickened leaflets, restricted motion, and severe stenosis (effective orifice area: 0.92 cm^2^, mean gradient: 41 mm Hg, maximum velocity: 4.46 m/s, aortic valve velocity time integral: 84.1 cm, aortic valve velocity ratio: 0.25, indexed effective orifice area: 0.35) compared with his prior TTE in 2017. His EGD was pertinent for an arteriovenous malformation (AVM) in the duodenal bulb, as shown in Figure 2. A send-out laboratory test for von Willebrand factor (VWF) activity taken at the end of his hospitalization was within normal limits at 78%.

## Management and Outcome

On presentation, the patient was transfused with 2 U of pRBC, and his AVM was clipped during the EGD. His symptoms and clinical status improved promptly, and his hemoglobin remained stable during the remainder of the hospitalization. He was diagnosed with HS, and a referral was placed to cardiology for valve-in-valve transcatheter AVR (TAVR) consideration.

Presently, several months after his hospitalization, the patient has not had any significant adverse medical events. On his most recent follow-up with his primary care provider, he indicated fatigue and weakness, although he denied dyspnea at rest or on exertion. He has an appointment with the cardiology team scheduled for the future.

## Discussion

The association between AS and GIB was first observed by Dr Edward Heyde in 1958; however, it took several decades to identify gastrointestinal AVM as the culprit lesions. Some studies estimate that up to 3% of patients with AS are affected by HS.^1^ Although the pathogenesis of HS is still debated, the current theory is that patients develop an acquired type IIA von Willebrand disease (VWD). VWF, namely high–molecular weight VWF, is necessary for effective platelet adhesion and aggregation in the primary hemostasis process, as well as factor VIII stabilization for secondary hemostasis. In patients with AS, the stenotic valve generates high shear-stress forces, promoting VWF proteolysis, classically by ADAMTS13, which results in the degradation of high–molecular weight VWF.

For patients with a high clinical suspicion of HS, the initial diagnostic work-up includes obtaining a complete blood count with differential as well as an iron, coagulation, and hemolysis panel. In order to identify and treat sources of GIB, EGD and colonoscopies are frequently conducted. Of note, the most common AVM site in the setting of HS is the small intestine, namely the jejunum, followed by the duodenum and the ileum, respectively. This is in contrast to the majority of AVMs, which most frequently occur in the cecum and ascending colon.^2^ Therefore, a negative endoscopic study does not necessarily rule out HS; under such circumstances, a capsule endoscopy or balloon enteroscopy may be warranted to fully assess the small bowel. The aortic valve is assessed by TTE to establish the severity of the stenosis; although most patients with HS have severe AS, the disease process can occur even with moderate AS,^2^ particularly in patients with high-output heart failure.

Assessing VWF levels is another diagnostic test commonly obtained to document the deficiency or dysfunction of VWF; nonetheless, prior studies have found that a significant subset of patients might have no biochemical evidence of acquired VWD through serum testing.^2^ This may suggest that VWF is not a sensitive marker for a diagnosis of HS. For instance, as patients with known VWD get older, laboratory normalization of VWF may occur, which can be seen in up to 43% of patients.^3^ Further, there is some evidence suggesting that pRBC transfusions induce VWF release from endothelial cells, resulting in a transient increase in VWF antigen levels.^4^ This observation is especially relevant because many patients with HS require pRBC transfusions. Overall, the diverse presentations and diagnostic uncertainties surrounding HS may partly explain why the mean time to diagnosis has been reported as nearly 2 years.^2^

After managing the initial, acute GIB, the definitive treatment for HS is AVR. This is evident from studies indicating that approximately 93% of patients who undergo AVR have complete resolution of their GIB, compared with 5% of those who undergo a surgical approach for hemostasis.^5^ At the biochemical level, such findings are attributed to a rapid recovery of acquired VWD after AVR, with the condition reversing in up to 85.9% of patients within 1 day of the procedure.^6^

In pursuing AVR, patients are offered diverse options, including surgical AVR (SAVR) and TAVR prosthetic valves. For HS patients with a SAVR, a retrospective single-center study found that those with a mechanical valve had a 50% increased risk of GIB recurrence, versus 15% of patients with a bioprosthetic valve.^7^ Although this difference was not statistically significant, bioprosthetic valves are often preferred in HS to mechanical valves, which strictly require warfarin use. For those who receive a TAVR, a mild or greater paravalvular leak has been associated with an increased risk of GIB recurrence.^8^ Interestingly, when such regurgitations are addressed with aortic valve balloon dilation, the acquired VWD often resolves within minutes.^8^ Collectively, when comparing SAVR and TAVR outcomes in HS, a national retrospective investigation found similar all-cause mortality between groups; however, it was noted that patients with TAVR had lower rates of stroke and blood transfusions, as well as a shorter hospitalization course.^9^ Additionally, a minimally invasive approach through TAVR is often preferred in this unique patient demographic given the association between HS, severe AS, and advanced age.

Although a comprehensive work-up was conducted that allowed us to eventually formulate a definitive diagnosis for our patient, there were some gaps and limitations in testing. For instance, an initial iron panel was not obtained before blood transfusion in the emergency department, which may have further supported blood loss anemia on presentation. Further, in addition to the VWF assay, a factor VIII activity level was not obtained. Lastly, given the significant anemia on presentation, the aortic valve gradient and velocity were likely overestimated; as such, outpatient follow-up echocardiography would be beneficial to better quantify the degree of AS.

## Conclusions

Our case report describes the unique presentation of Heyde syndrome approximately 1 decade after bioprosthetic AVR. For this patient, it was his second presentation within 1 year for symptomatic, severe anemia. Although he did not notice frank melena or hematochezia, a detailed history found a change in his stool to a darker and stickier consistency. His EGD subsequently discovered an AVM, and TTE was consistent with severe bioprosthetic aortic stenosis. Clinicians with a strong suspicion for Heyde syndrome and no observed AVM on EGD or colonoscopy should consider pursuing capsule endoscopy or push enteroscopy to fully assess the small bowel. Additionally, while this patient's VWF level was within normal range, there may be other confounding factors to this result, such as age and recent blood transfusion, which should not in isolation rule out a diagnosis of Heyde syndrome.

## Funding Support and Author Disclosures

The authors have reported that they have no relationships relevant to the contents of this paper to disclose.

## Appendix

Video 1 Transthoracic echocardiography clip in the parasternal long-axis view.
